# Walter's Duiker (*Philantomba walteri* Colyn, 2010): A Literature Review on a Recently Identified and Potentially Vulnerable Species

**DOI:** 10.1002/ece3.70473

**Published:** 2024-11-20

**Authors:** Eltsine M. C. Sahgui, Simon Lhoest, Davy Fonteyn, Kasso Daïnou, Johan Michaux, Cédric Vermeulen

**Affiliations:** ^1^ Gembloux Agro‐Bio Tech., University of Liège Gembloux Belgium; ^2^ École Régionale d'Aménagement et de Gestion Intégrés Des Forêts et Territoires Tropicaux (ERAIFT) Kinshasa Democratic Republic of the Congo; ^3^ CIRAD, UPR Forêts et Sociétés Campus International de Baillarguet Montpellier Cedex 5 France; ^4^ Forêts et Sociétés Univ Montpellier, CIRAD Montpellier France; ^5^ National Institute for Health and Disability Insurance (NIHDI) Brussels Belgium; ^6^ Conservation Genetics Laboratory University of Liège Liège Belgium

**Keywords:** antelopes, Bovidae, Dahomey‐gap, fruit, hunting, Walter's duiker

## Abstract

Based on recent taxonomic and molecular tools, the Walter's duiker (*Philantomba walteri* Colyn, 2010), endemic to the Dahomey Gap in West Africa, has been recognized as a new species in 2010. This species is largely hunted and may already be threatened by extinction. This review paper aims to synthesize the current knowledge on this species, covering its taxonomy, morphology, biology, ecology, diet, seed dispersal role, reproduction patterns, activity rate, parasitology, spatial distribution, habitats, population densities, and ongoing human pressures. We carried out an exhaustive literature search using nine databases, going through 1200 initial references to finally retain a total of only 11 research articles mentioning Walter's duiker. Very few publications exist on the species due to its recent discovery. Existing studies focus on feeding, parasitology, and hunting pressure. Walter's duiker distribution range extends over Togo, Benin, and Nigeria. The species is hunted in a large part of its range, including in the reserves that are supposed to protect it. We synthesize the biological information related to the Maxwell's duiker as well as the Walter's duiker, as historically, these two species have been confounded and are morphologically similar. Our synthesis also highlights the scientific gaps for a better understanding of the biology of this species, and it proposes priority themes for future research. Priority should be given to studying the diet composition of Walter's duiker by analyzing rumens and feces, its role in seed dispersal and forest regeneration, its home range and activity rate, and the estimation of its abundance. All these information together would allow to thoroughly assess the species status and contribute to its conservation.

## Graphical abstract

The Dahomey Gap, a dry corridor, divides the African tropical rainforests into distinct blocks. With an annual rainfall below 1200 mm, it hosts savannas, crops, and isolated fragments of gallery forests, swamp forests, and semi‐deciduous forests. Despite strong human influence, the discovery of Walter's duiker in 2010—a small antelope intermediate between the blue duiker and Maxwell's duiker—highlighted its unique endemism. However, hunting pressure, habitat loss, and climate change threaten its existence in this region. Eleven studies aimed at its conservation cover species description, identification during wildlife surveys, meat safety risks, gastrointestinal parasites, and hunting methods. Yet, crucial scientific data remains incomplete, such as population size, diet, behavior, reproduction, habitat range, and life cycle. Addressing these gaps promptly is essential for the sustainable preservation of the species.

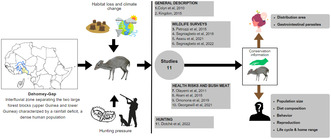

## 1 Introduction

Environmental changes, and climate in particular, have largely shaped the diversity of species found on Earth today. The major climatic oscillations of the Pleistocene, particularly from 1.05 Ma onwards, caused cycles of fragmentation and expansion of tropical forests (Dupont et al. 2001; Miller and Gossling 2014), resulting in the spatial and the genetic isolation of many taxa over long periods. In sub‐Saharan Africa, the last major glaciation ended around 10,000–11,000 years BP. It was only after this period, following the return of a warmer, wetter climate, that the dense forest gradually recolonized its current area, encouraging new contacts between previously separated animal and plant populations. However, around 3500–4500 years BP, a fairly brutal climatic dry spell occurred and persisted in the coastal zone from eastern Ghana to the eastern limits of Benin, slowing forest recolonization and giving way to the “Dahomey Gap” (Salzmann and Hoelzmann 2005), a corridor of forest‐savanna mosaics of approximately 200 km width (between 0° and 3° East) with only 1000–1200 mm of cumulative rainfall per year. It comprises vast tracts of herbaceous or cultivated land mixed with a network of fragmented forest patches of varying size (from 2 ha for the Lébé forest in Togo to more than 204,218 ha for the Mono Delta Biosphere Reserve) (Salzmann and Hoelzmann 2005; Demenou, Doucet, and Hardy 2018; Segniagbeto et al. 2022);

In addition to climatic conditions, these patches of forest have also been shaped by human pressures (notably agricultural development, charcoal production, and timber exploitation). For example, all the small patches of forest identified by Segniagbeto et al. (2022) were previously one large patch.

These climatic conditions maintained genetic isolation between populations and may have led to the speciation of taxa now recognized as endemic to the Dahomey Gap, eminent examples being the red‐bellied monkey (*Cercopithecus erythrogaster* Gray 1866), the Togo slippery frog (*Conraua derooi* Hulselmans 1972) and Walter's duiker (*Philantomba walteri* Colyn, 2010).

The latter, recognized as a species in 2010, had long been assimilated to its forest cousin, the Maxwell's duiker (Colyn et al. 2010). However, modern taxonomic classification methods, including DNA analyses, led to confirm the specificity of the Walter's duiker. Thus, before this recognition, the information concerning Walter's duiker would be amalgamated with that of Maxwell's duiker, hence the need to dissociate them clearly.

The aim of this review paper is to synthesize the existing knowledge of this little‐known and probably already threatened duiker species, focusing on different aspects allowing to propose appropriate management and conservation strategies: (1) taxonomic and morphological description; (2) ecological and biological characteristics, including diet, seed dispersal role, reproduction patterns, activity rate, and parasitology; (3) spatial distribution, including habitats and population density; and (4) ongoing human pressures on its populations.

## 2 Methods

**FIGURE 1:**
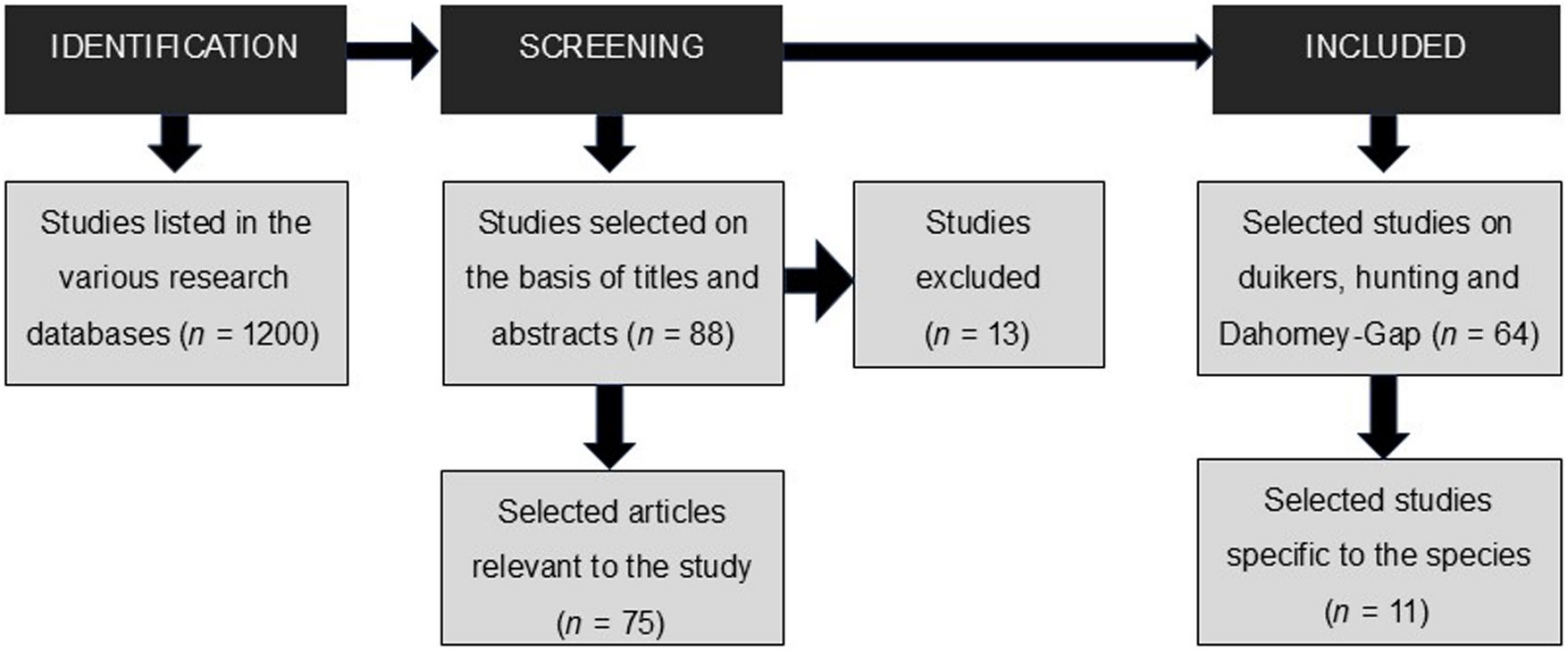
Flow chart for the selection of articles included and excluded from the analysis.

**FIGURE 2:**
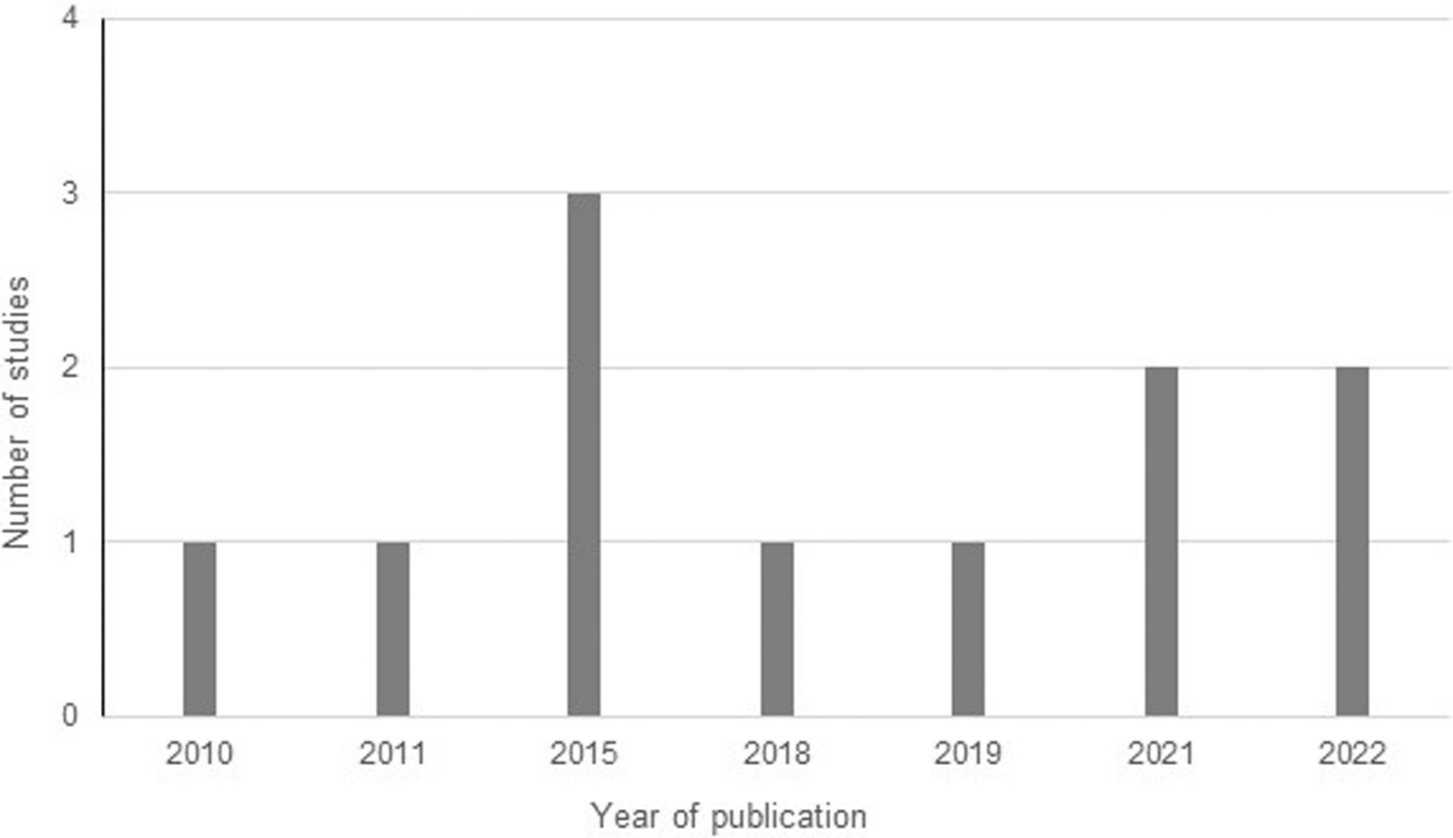
Number of studies on the Walter's duiker from 2010 to 2023.

A literature search was carried out using Publish or Perish software (Harzing 2016), using the following databases: Google Scholar, Scopus, BASE (Bielefeld Academic Search Engine), ORBi (Open Repository and Bibliography of the University of Liège), Crossref, OpenAlex, PudMed Semantic Scholar and Web of Science (Moral‐Muñoz et al. 2020). The following keywords were used in French and English: “Walter's duiker”, “Maxwell's duiker”, “taxonomy”, “morphology”, “diet”, “seed dispersal”, “reproduction”, “parasitology”, “distribution”, “habitat”, “count”, “density”, “pressure”, “Dahomey‐Gap”, “conservation”, and “hunting”. The scientific names of the two species, “*Philantomba walteri*” and “*Philantomba maxwelli*”, were also included in the literature search. Combining keywords in the various databases yielded almost 1200 references. From these results, a selection of publications was made based on titles and abstracts, which initially resulted in 88 articles being retained. After reading the full texts, 13 articles were rejected either because they were not informative or because they did not focus on duikers. We finally retained 75 articles with a maximum age limit of 13 years for information regarding the ecology of the species as the time since the species was discovered, and no time threshold for general information on duikers, hunting, and the Dahomey Gap. Of all these references, only 11 mentioned Walter's duiker. The flowchart for selecting the articles included and excluded from the analysis (Figure 1) and the number of studies on Walter's duiker from 2010 to 2023 (Figure 2) are presented below.

## 3 Taxonomy and Morphology

Duikers are antelopes from the family Bovidae, subfamily Cephalophinae, grouped into three main genera: *Sylvicapra* Ogilby, 1837, *Cephalophus* C.H. Smith, 1827 and *Philantomba* Blyth, 1840 (Jansen van Vuuren and Robinson 2001; Grubb and Groves 2005; Colyn et al. 2010). The taxonomic status of the genus *Philantomba* has undergone recent substantial changes, following phylogenetic analyses using mitochondrial and nuclear DNA sequences and craniometric measurements. Until 2010, the genus *Philantomba* was considered a synonym, or at best, a subgenus of the genus *Cephalophus*, and only two species were recognized: *Philantomba maxwelli* and *P. monticola*. However, Grubb and Groves (2001) validated the separation into two distinct genera, and their cladistic analysis places *Philantomba* as a “sister” genus to *Sylvicapra* rather than *Cephalophus*. Independently, Jansen van Vuuren and Robinson (2001) also confirmed this proximity between the two genera using a genetic approach based on evidence from mitochondrial DNA sequences and fluorescence in situ hybridization.

*Philantomba* are small duikers, their head and body measuring less than 75 cm in length. They are characterized by a thick layer of hair on the forehead, which is darker and browner than the face itself. There is usually (but not always) a red or buff stripe above each eye (Groves 2013). The coat does not change with age; the hairs are uniform in color, usually a shade of brown, darker on the back and flanks, and there is often a clear transition between dark and light tones on the hip. As with *Sylvicapra*, the tail is uniformly haired and not bushy. The skull is small and delicate, relatively wide in the cranium and the frontal region, and abruptly narrowed on the rostrum, so that the anterior width of the nasals is only half that of the frontonasal suture. The occipital plane and the paraccipital processes are oblique and somewhat tubular, and their dorsal edge protrudes somewhat beyond the plane of the frontals (all these features are the same as in *Sylvicapra*). The preorbital fossa is very wide, but shallow (Groves 2013). The horns are very small, delicate, and inverted at the tip; in females, they are very small and often absent. In the pelvis, the lengths of the ilium and ischium are roughly equal, as in *Sylvicapra*. In addition to these diagnostic features, other characters (which are sometimes also found in members of the genus *Cephalophus*), the absence of inguinal glands, the lack of a true crest and the tendency for the neck hairs to become inverted (Grubb and Groves 2001).

The species *Philantomba walteri* had not previously been described or named. Nor was it distinguished from Maxwell's duiker. It was only following the work of Colyn et al. (2010) that this species was differentiated and described morphologically. Thus, from the morphological description of Colyn et al. (2010) and Kingdon (2015), *Philantomba walteri* is intermediate in size between the “large” *Philantomba maxwelli* and the smaller *Philantomba monticola*. Externally, the new species resembles *Philantomba maxwelli* due to its relatively long tail (> 15 cm), large pedal gland, striking superciliary line, the absence of a strongly marked break on the hips between the dark rump, light flanks, and lower hips. The head is clearly less massive, and the overall silhouette is slenderer. From a craniometric point of view, *Philantomba walteri* can be distinguished from the other two *Philantomba* species by its much smaller nasal constriction and cranial height. The molecular data indicate that *P. walteri* is a distinct evolutionary unit compared with its congeners *P. maxwelli* and *P. monticola*.

**FIGURE 3:**
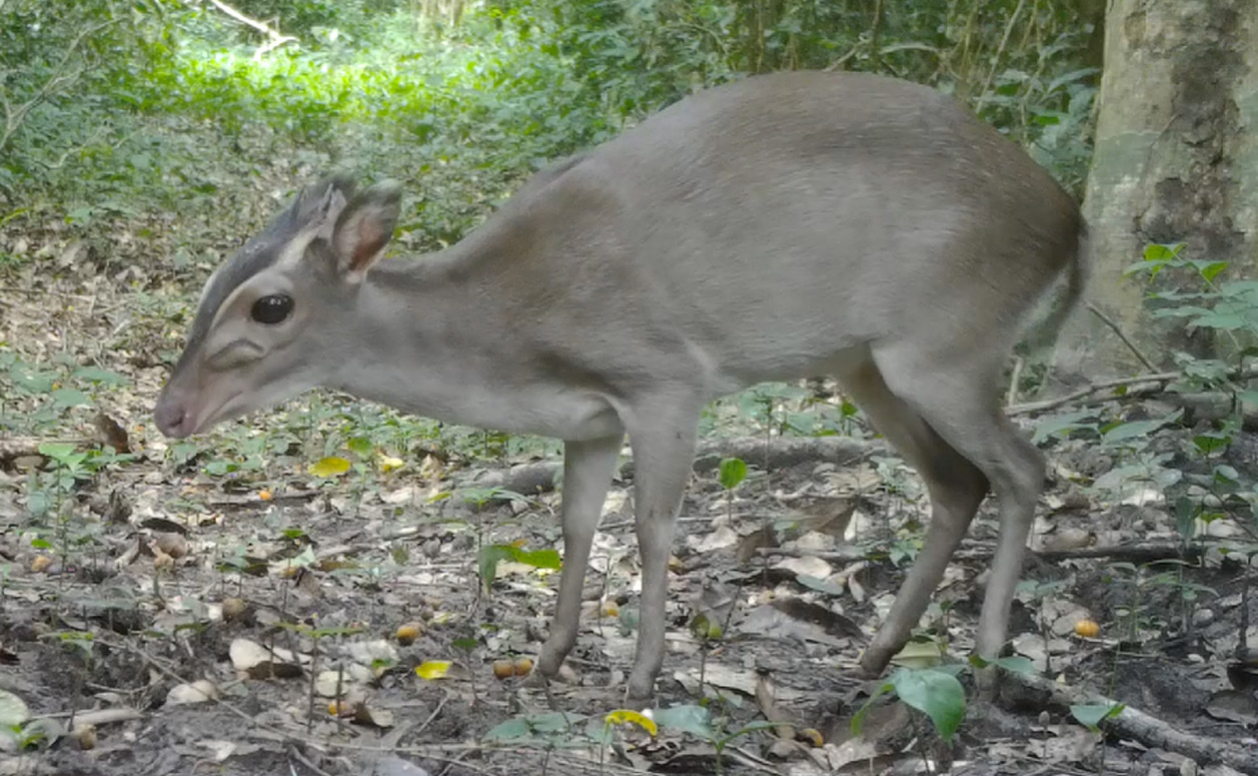
Illustration of the Walter's duiker species (*Philantomba walteri* Colyn, 2010).

The morphological illustration of Walter's duiker is shown in Figure 3 (from photographic traps in the lama classified forest in Benin in 2023).

## 4 Ecological and Biological Characteristics

### 4.1 Diet

The diet of most duikers has been documented. They are recognized as primary consumers, with a diet consisting of up to 90% of fruit plus leaves (Feer 1989; Houngbégnon et al. 2019). In their natural habitat, duikers also feed on animal proteins. In captivity, however, they are sometimes fed on dog food as a supplement (Burton and Burton 2002). They are very active and need to eat large quantities of food (fruit) to maintain their energy levels (Burton and Burton 2002).

As Walter's duiker has long been confused with Maxwell's duiker, we will mention the observations available on the diet of the latter. Maxwell's duiker are mainly frugivores, but they also eat leaves and fungi, and may depend on leaves in times of fruit shortage (Nett and Newing 2013). The two most comprehensive feeding studies are by Hofmann and Roth (2003) and Wilson (2001). Hofmann and Roth (2003) examined 139 stomachs from the Toumodi bushmeat market in Côte d'Ivoire. A total of 78 different fruit species were found in the stomach contents of the Maxwell's duikers studied, with a preference for *Nauclea latifolia*, *Ficus capensis*, *Canthium vulgare*, *Blighia sapia*, *Griffonia simplicifolia*, *Alchornea cordifolia*, *Phoenix reclinata*, and *Spondias mombin*. Wilson (2001) examined 250 stomachs from bushmeat markets in Ghana and recorded 33 species of fruit, including *Ficus* spp., *Solanum* spp., *Blighia* spp., *and Uvaria* spp. All the consumed fruit came from cultivated lands, secondary forests, or savanna. In the semi‐deciduous forests of eastern Côte d'Ivoire, hunters named the fruits of *Ricinodendron heudelotii*, *Strombosia glaucescens*, *Trichilia monadelpha* (= *T. heudelotii*), *Ficus exasperata*, *Celtis adolfi‐fridericii*, and *Ceiba pentandra* as their main food sources (Nett and Newing 2013). Newing (1994, 2001) listed 26 fruit species found in eight stomach samples collected in Côte d'Ivoire, plus two other species consumed during direct observations. Hofmann and Roth (2003) and Wilson (2001) both recorded invertebrate remains in the studied stomachs, notably ants. Maxwell's duiker is thought to follow groups of colobus monkeys to take advantage of fruit that has fallen to the ground (Nett and Newing 2013). Indeed, the only available study on the diet of the Walter's duiker in Benin (Dotché et al. 2022) is based on surveys of hunters, farmers, and water and forestry officers, that is, observations based on what the actors say, which are often considered to be biased or incomplete. Nevertheless, this study shows that Walter's duiker consumes around 29 plant species from 20 families, mainly Euphorbiaceae (Dotché et al. 2022). Of these plants, eight types of organs are consumed, including leaves (29 species, 100% of respondents), bark (72%), fruit (27%), and stems (24%), which were the most popular organs. This study suggests that leaves are the main food of this duiker, unlike all the other more frugivorous species (Dotché et al. 2022). This surprising finding and the paucity of available data call for further research into the feeding behavior of Maxwell's and Walter's duikers. In view of the particular methodology used, it can be concluded that the knowledge of the species' diet is not yet precise and that of Maxwell's duiker cannot be generalized to the Walter's duiker. Further researches are required.

### 4.2 Seed Dispersal

Dispersal plays a crucial role in the population dynamics of plant species (Treep et al. 2021). With a diet that is 90% frugivorous (Houngbégnon et al. 2019), duikers make a major contribution to the stability of forest ecosystems by dispersing the seeds of fruit they eat (Dorst 1991; Charles‐Dominique 2003; Kortenhoven 2009). They also complete the range of dispersers of plant species whose regeneration is deficient in mature forests (Daïnou et al. 2012; Duminil et al. 2016). However, the potential for dispersal depends not only on the size of the duiker, but also on the size of the seeds. The smallest species of duikers do not swallow fruits larger than 3 cm in diameter, whereas the largest can swallow fruits up to 6 cm in diameter (Dubost 1984). Thus, small seeds of *Solanum verbascifolium* L. and *Musanga cecropioides* R.Br. ex Tedlie can escape the process of mastication and digestion and end up in the feces of the black duiker, *Cephalophus niger* Gray (Alexandre 1982). Another possibility for seed dissemination by duikers is their ability to spit out seeds far from seed sources during their rumination process (Gautier‐Hion et al. 1985). According to Feer (1995), the seeds that duikers may reject during rumination can be determined either by analyzing their rumen contents or by observation in captivity.

Houngbegnon et al. (2022) showed in central African forests that fertile seeds of certain commercially valuable species such as *Milicia excelsa*, *Nauclea diderrichii*, and *Erythrophleum suaveolens* are present in the feces and rumens of certain duiker species (*Cephalophus silvicultor*, *Philantomba congica*, *C. callipygus*, and *C. castaneus*). For other species, such as *Erythrophleum suaveolens*, seeds can only be expelled by regurgitation (Houngbegnon et al. 2022). The role of Walter's duiker in seed dispersal, through defecation or rumination, is still unknown to this day.

### 4.3 Reproduction

Knowledge of the duiker reproduction is still incomplete (Houngbégnon et al. 2019). Duikers live in monogamous couples or sometimes in polygamous groups (Newing 1994), with or without offspring, in a common territory. *Philantomba*, on the other hand, live in stable couples that maintain permanent bonds with each other (Dubost 1980). Mating is generally preceded by courtship, during which the male pursues the female. At this time, males can be very aggressive towards other animals (Castello 2016). However, the gestation period for species in the *Philantomba* genus remains open to debate. For the Maxwell's duiker, the durations vary according to the authors, from 120 days (Aeschlimann 1963), 167 days (Mentis 1972), 188 days (Kadjo 2000) to 205 days (Dittrich 1972; Wilson 2001). There is no information available strictly for the Walter's duiker. Knowledge of sexual maturity is also incomplete and sometimes contradictory for the genus *Philantomba*. In captivity, *Philantomba monticola* males reach sexual maturity at 9 months and females at 6–17 months (Böhner, von Volger, and Hendrichs 1984). Wilson (2001) notes that females *Philantomba monticola* generally become sexually mature before 13 months, when males reach sexual maturity between 11 and 14 months. As for the Maxwell's duiker, Wilson (2001) recorded four captive females conceiving between 8 and 12 months, and the earliest successful mating by a male at 10 months. However, Kadjo (2000) did not record the first estrus until 18 months and observed that females became receptive again 3–5 days after giving birth.

Most of the data on duiker reproduction come from studies of animals in captivity, and it is therefore difficult to generalize such observations to animals living in the wild. Everything remains to be studied concerning these aspects for the Walter's duiker.

### 4.4 Activity Rate

There is no information on the activity rate of the Walter's duiker. The information gathered during camera trap counts does not mention the species' activity rate. However, with regard to Maxwell's duiker, a study using camera traps in Taï National Park in Côte d'Ivoire indicates that duikers were rarely filmed during the hours of darkness, and none were filmed between midnight and 6:00 am. Detection frequency increased steadily after 6:00 to peak between 6:30 and 7:00 and remained relatively high until 9:30, after which it decreased slightly and remained relatively low until 16:30, then increased again and remained high until 18:00, before gradually decreasing until 19:00 (Howe et al. 2017). Thus, information relating to the species' activity rate remains to be documented.

### 4.5 Parasitology

Disease is one of many factors threatening the existence of wild animals, some of which are infectious parasitic diseases originating from gastrointestinal parasites (Singh, Shrivastav, and Sharma 2009; Thawait, Maiti, and Dixit 2014). Parasites play an important role as mechanisms for regulating the population dynamics of species within an ecosystem (Tompkins et al. 2002; Begon et al. 2006). Parasitic infections and their complications are major threats to wild animal populations and can act as agents of extinction (Harvell et al. 2002; Jog and Watve 2005). Parasitological surveys of bushmeat markets in Nigeria reveal that Walter's duiker is host to nine parasites: Strongylidae, *Cooperia* spp., *Protostrongylus* spp., *Eimeria* spp., *Toxocara* spp., *Haemonchus* spp., *Fasciola* spp., *Strongyloides* spp. and *Paramphistomum* spp. (Omonona, Ademola, and Ayansola 2019). These parasites can contaminate the environment and represent a source of disease transmission to domestic animals and humans (Omonona, Ademola, and Ayansola 2019). However, the effects of these parasites on the host and their ability to establish themselves in other animals still need to be studied thoroughly.

It can be concluded that a few parasites are known for the species, but this is only the result of a single study. So, complementary studies should be carried out to complete the existing knowledge on this subject, particularly the direction of infections (from duiker to domestic animals or to humans, or vice versa).

## 5 Geographical Distribution and Habitat

**FIGURE 4:**
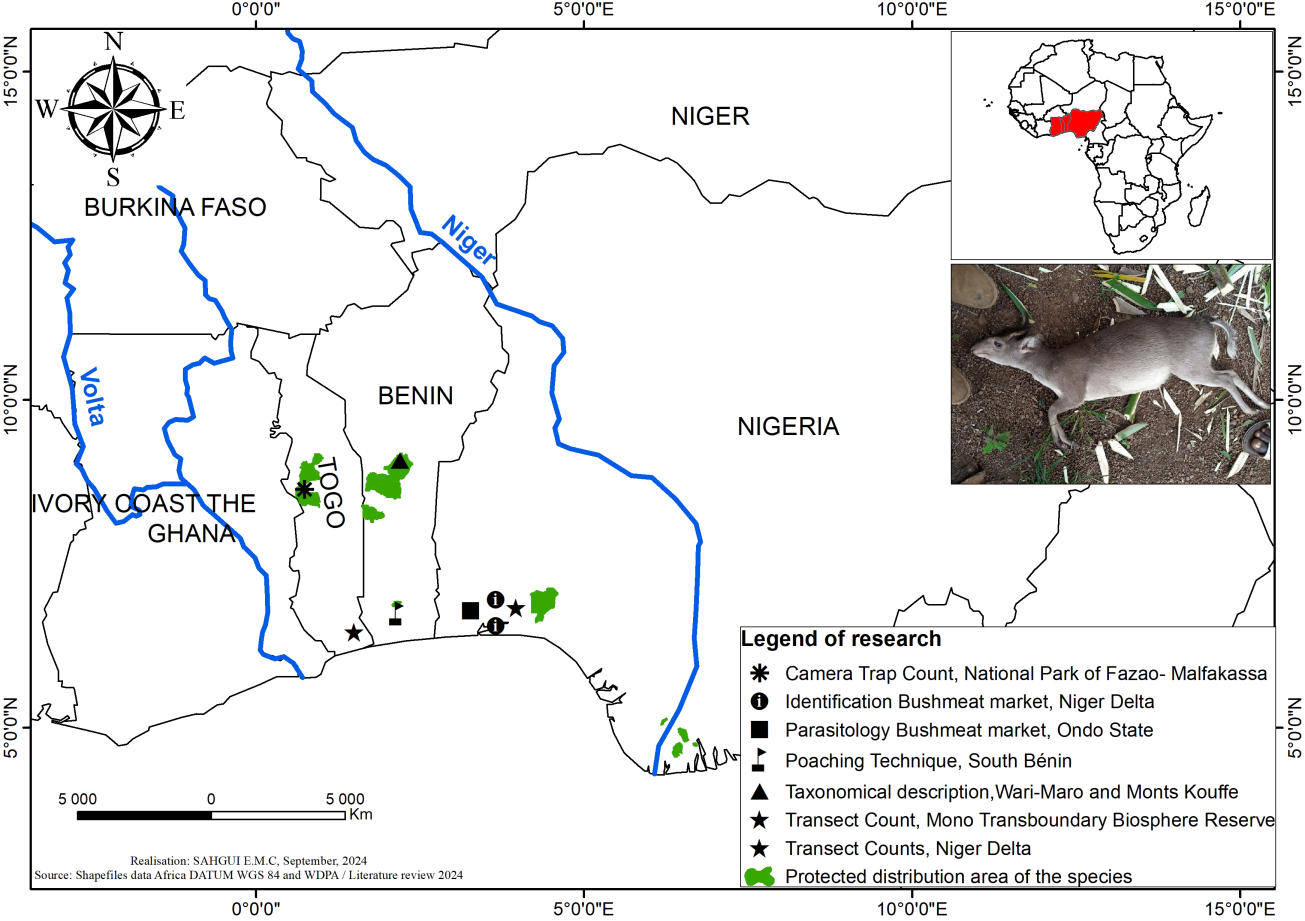
Range of Walter's duiker (*Philantomba walteri* Colyn, 2010).

The geographical distribution of the Walter's duiker extends across the Dahomey Gap, from Benin to Togo and as far as the Niger Delta in Nigeria (Figure 4).

### 5.1 Habitats

The habitats of Walter's duiker are not fully known yet, but Colyn et al. (2010) report that the specimens observed in their study came from the Beninese forest massifs of Agoua, Wari‐Maro, and Monts Kouffè (Colyn et al. 2010). The presence of the species has also been reported in the Lama forest regions (Colyn et al. 2010), where the vegetation consists in a natural dense forest (part of the central core), forest plantations of *Tectona grandis* and *Gmelina arborea*, and mosaics of crops, fallow land and shrub savannas on riparian land (Folahan et al. 2018). Moreover, according to Kingdon (2015), Walter's duiker uses the same habitat as Maxwell's duiker, that is, rainforest galleries and relics and wet savannas. In Togo, it has been reported in southeastern forests of Amévo, Fonta, Zogbévé, Mambui, Dougbanavé and Avélébé, characterized by dense thickets of *Morelia africana, Drepanocarpus lunatus, Qlchornea cordifolia, Paullinia pinnata, Pterocarpus santalinoides*, and *Phoenix reclinata* (Segniagbeto et al. 2022). It is also found in the Fazao‐Malfakassa park (Assou et al. 2021), where altitude varies between 300 and 800 m (Atsri et al. 2018; Segniagbeto et al. 2017). This park is located at the boundary between the Sudanese ecological zone and the Guinean ecological zone and is characterized by dense semi‐deciduous forest vegetation and scattered wooded savannas (Assou et al. 2021). Clearly, Water's duiker, like Maxwell's duiker, appears to adapt to a wide range of environments, from mature forests to degraded areas dominated by herbaceous plants such as *Chromolaena odorata* (Nett and Newing 2013).

In Nigeria, Walter's duiker was recorded in the bushmeat market of Bayelsa in the Niger Delta (Akani et al. 2015). In the same Bayelsa region, it has been observed in the Taylor Creek, Nun River, Edumanom, Egbedi Creek, and Upper Orashi forest reserves in the Rivers region (Petrozzi et al. 2015). These forest reserves are characterized by a diversity of vegetation, including seasonally flooded swamp forests along the River Niger, mosaics of plantations and forests, freshwater swamp forests, and tropical rainforests with seasonal flooding and multi‐layered vegetation (Petrozzi et al. 2015). This duiker is also found in the bushmeat markets of Omagwa, Oyigbo, and Mbiama in Rivers State (Georgewill et al. 2021). It is also found in Ondo State (Nigeria) where studies have been carried out by Omonona, Ademola, and Ayansola (2019) on the prevalence of gastrointestinal parasites of the species. These states are characterized by rainforest, grassland, swamp, and mangrove vegetation.

Fasona et al. (2024) assessed the impact of habitat modifications on large mammals in the Omo Shasha reserves in Nigeria and identified Maxwell's duiker. The Shasha reserve is characterized by pockets of degraded natural forest to which are added plantations of various species, including *Gmelina arborea*, *Tectona grandis*, *Terminalia* spp., *Pinus* spp., and *Nauclea diderichii*, while the Omo reserve is characterized by semi‐deciduous mixed rainforests, most of which are degraded (Jimoh et al. 2012). These forests are complemented by *Gmelina arborea* plantations (Jimoh et al. 2012).

These protected areas lie within the estimated range of Walter's duiker. Previous studies in Nigeria have used genetic analysis of bushmeat and could, therefore, help to identify the species with greater certainty.

In conclusion, the species' range appears to be known, although this latest study by Fasona et al. (2024) still causes some confusion. However, questions remain as to the distribution of the species in part or all of the Dahomey‐Gap. Nevertheless, it appears that the species uses habitats modified by man, such as plantations and crops. However, observations on its habitat preferences remain incomplete.

### 5.2 Counts and Density

The few studies mentioning *P. walteri* were generally aimed at determining the species richness of the study areas. No study to determine the specific abundance of this species is yet available. However, species management requires at least a knowledge of certain key population parameters, such as changes in numbers over time (Bouché 2008). The only inventory data on the Walter's duiker populations is available for certain protected areas in Togo. In Fazao‐Malfakassa National Park, a camera trap survey showed a detection rate for Walter's duiker of 0.022 captures per 100 days of cumulative inventories, making it one of the least detected species in the park (Assou et al. 2021). During a count of ungulates in the Mono Transboundary Biosphere Reserve in Togo, the species was directly observed 30 times using the opportunistic transect counting method along forest paths (Segniagbeto et al. 2018). This count was carried out using the strip transect method. A total of 255.1 km with a total width of 130 m was covered, corresponding to 3316.3 ha and representing 13% of the total area of the Togodo reserves. The Togodo Reserve comprises the Togodo South National Park and the Togodo North Wildlife Reserve, both part of the Mono Delta Transboundary Biosphere Reserve.

Currently no studies have attempted to estimate the density of Walter's duiker in its distribution range. Conversely, several studies mention this parameter for *P. maxwelli*, making it a common taxon (Wilson 2001) with estimated densities ranging from 15 to 30 individuals/km^2^ in mature logged forests in the Gola National Park in Sierra Leone (Davies 1991). It is also the most common duiker in Côte d'Ivoire, where Newing (1994, 2001) recorded densities of 79 individuals/km^2^ in a mixed agricultural area near the Taï National Park and 63 individuals/km^2^ in lightly logged primary forest with minimal hunting within the park. Howe et al. (2017) indicate a lower density ranging from 11 to 17 individuals/km^2^ in the same park. Elsewhere in Côte d'Ivoire, and again for Maxwell's duiker, Nett (1999) recorded a density of 16–20 individuals/km^2^ in a heavily logged semi‐deciduous forest.

Thus, counting the Walter's duiker and calculating its abundance or density as a function of habitat remain aspects that need to be documented.

## 6 Human Pressures

In West Africa, large‐scale bushmeat extraction combined with the dramatic alteration of the natural forest cover (Achard et al. 2002; Fasona and Omojola 2009) has led to serious declines in large mammal populations in recent decades, including in protected areas (Craigie et al. 2010). Duikers are no exception to this trend and are among the most hunted forest animals (Wilkie and Carpenter 1999; Csuti 2010; Kümpel et al. 2010; Macdonald et al. 2012).

Duikers represent an important source of food and income in all forested regions (Juste et al. 1995; Muchaal and Ngandjui 1999). They are recognized as the primary products of hunting and as such account for a significant proportion of the harvest in terms of both numbers and biomass (Hart 2000; Mockrin 2009; Yasuoka et al. 2015). As a consequence, most species belonging to this group are considered endangered due to the ongoing decline in their populations across sub‐Saharan Africa (Ghassemi‐Khademi and Hamidi 2019).

Walter's duiker is one of the most hunted species in the Asejire roadside bushmeat market in south‐west Nigeria (Olayemi et al. 2011). It can account for up to 70% of the number of individuals among various antelope species hunted in southern Nigeria in the Omagwa, Oyigbo, and Mbiama areas of Rivers State (Georgewill et al. 2021). In Benin, it is hunted mainly with locally made rifles, metal traps, snares, and nets (Dotché et al. 2022). The effects of hunting are already being felt in southern Benin by the people living around the Lama classified forest. They are reflected in the rarity of several species, including the Walter's duiker, in the patches of forest around the Lama classified forest (Djagoun et al. 2022).

In Nigeria, the selling price of a Walter's duiker varies from region to region. In the south, it varies from 6000 to 11,000 Naira, corresponding to 11.90–21.81 euros (Georgewill et al. 2021). In the south–west, the price is around 4500 Naira or 28.30 euros (Olayemi et al. 2011). In Benin, in addition to the meat, various other parts are sold, including the skin, horns and urine for use in traditional medicine. A whole animal sells for around 17 euros each (Dotché et al. 2022). A few studies on hunting practices for this species are therefore available, but they remain limited. However, they show that the species is hunted in a large part of its range, including in the reserves that are supposed to protect it.

## 7 Conclusion

At the end of this literature review, we can conclude that the Walter's duiker is a recently described species, endemic to its range, which extends over three countries: Togo, Benin, and Nigeria. Over the last decade, it has appeared in several studies conducting animal surveys and focusing on hunting practices. The various parasites contained in its meat have been described for Nigeria.

This assessment, based on only a few available references, highlights the need for more studies on this antelope species to better address conservation issues. The species' recent discovery and the lack of interest shown in the medium and small fauna can explain this lack of study. It is essential to encourage and promote studies on the following priority areas: (i) the composition of the species' diet; (ii) its role in seed dispersal and forest regeneration; (iii) its home range and rate of activity; and (iv) estimates of its abundance and spatial distribution. This information could certainly help to determine the potential decline or otherwise of Walter's duiker, which is already vulnerable due to hunting pressure and the reduction of its habitats.

## Author Contributions

**Eltsine M. C. Sahgui:** conceptualization (equal), methodology (equal), writing – original draft (equal), writing – review and editing (equal). **Simon Lhoest:** conceptualization (equal), supervision (equal), writing – review and editing (equal). **Davy Fonteyn:** writing – review and editing (equal). **Kasso Daïnou:** writing – review and editing (equal). **Johan Michaux:** supervision (equal), writing – review and editing (equal). **Cédric Vermeulen:** supervision (equal), writing – review and editing (equal).

## Conflicts of Interest

The authors declare no conflicts of interest.

## Data Availability

The authors have nothing to report.
